# High diversity and distribution of ticks and tick-borne diseases in Sudan emphasizes the need for implementing cost-effective community One Health strategy

**DOI:** 10.3389/fmicb.2026.1790610

**Published:** 2026-05-19

**Authors:** Nouh Saad Mohamed, Ayman Ahmed

**Affiliations:** 1 Pan-Africa One Health Institute (PAOHI), Kigali, Rwanda; 2 Sennar Malaria Training and Research Centre, Sennar, Sudan; 3 Institute of Endemic Diseases, University of Khartoum, Khartoum, Sudan

**Keywords:** One Health, tick-borne diseases, ticks, vector control, vector distribution, vector surveillance, zoonoses

## Abstract

Ticks and tick-borne diseases (TBDs) represent a growing challenge to both animal and human health in Sudan. However, the absence of consolidated nationwide data limits accurate characterization of tick fauna, monitoring of shifts in species distribution, and detection of invasive species. This knowledge gap not only constrains understanding of vector ecology but also weakens efforts to link tick distribution with disease occurrence, thereby undermining targeted control strategies. Livestock TBDs, including theileriosis, babesiosis, anaplasmosis, and lumpy skin disease, cause substantial economic losses by reducing productivity, threatening food security, and hindering national development. In parallel, zoonotic infections such as Crimean-Congo hemorrhagic fever, Q fever, borreliosis, ehrlichiosis, rickettsioses, and potentially tularemia pose underrecognized public health risks. These infections are frequently misdiagnosed or overlooked due to limited diagnostic capacity and the predominance of malaria in the differential diagnosis of febrile illness. The persistence and spread of TBDs in Sudan are driven by interconnected factors, including high livestock mobility, widespread presence of competent vectors, weak veterinary and public health infrastructure, and inadequate surveillance systems. Addressing this multifaceted burden requires integrated approaches that combine improved vector control, expanded diagnostic capacity and surveillance, vaccination where available, and strengthened regional cross-border collaboration. This review synthesizes current knowledge on ticks and TBDs in Sudan, highlights critical gaps, and emphasizes the need for One Health strategies to reduce TBD transmission, protect livelihoods, and safeguard public health.

## 1 Introduction

Ticks are hematophagous arthropods belonging to the order Ixodida, which comprises three families: Argasidae (soft ticks), Ixodidae (hard ticks), and Nuttalliellidae (Barker and Murrell, 2004; Estrada-Peña, 2015). Each family includes species of considerable medical and veterinary importance, except Nuttalliellidae, which is rarely encountered and represented by a single species, *Nuttalliella namaqua* (Barker and Murrell, 2004). Ticks are important vectors of a wide range of infectious agents, including protozoa, bacteria, and viruses, many of which cause severe diseases in humans and animals (Labuda and Nuttall, 2004; Bakheit et al., 2012; Shi et al., 2018).

Ticks and tick-borne diseases (TBDs) impose a substantial burden on human health as well as on the health and productivity of animals, thereby challenging socioeconomic stability and growth. These impacts arise from mortality, reduced meat and milk production, and the costs associated with treatment and control measures (Uilenberg, 1992; Godfrey and Randolph, 2011). Globally, TBDs are increasingly recognized as emerging and re-emerging threats, driven by climate change, land-use and land-cover changes, population growth and mobility, ecological disruption, and more frequent human–animal interactions (Estrada-Peña et al., 2012; Mansfield et al., 2017; Choi and Lee, 2025).

Sudan, the third-largest country in Africa, encompasses diverse ecological and climatic zones, ranging from desert and semi-desert in the north to savannah in central regions and tropical forests in the south (ElGhali and Hassan, 2012; Mohamed et al., 2019). These varied environments provide favorable habitats for a wide diversity of tick species, supported by the abundance of domestic and wild animal hosts (ElGhali and Hassan, 2012; Altahir et al., 2022). The Sudanese economy is heavily dependent on livestock, with millions of cattle, camels, sheep, and goats contributing to food security, livelihoods, and export earnings. However, this sector remains under constant threat from tick infestations and associated diseases, which limit productivity and impose significant economic burdens on pastoralist and agro-pastoralist communities (Hurtado and Giraldo-Ríos, 2018; Salim et al., 2023; Makwarela et al., 2025). In addition, many TBDs pose increasing risks to public health, particularly in rural areas where human–animal contact is frequent and access to healthcare is limited (Bakheit et al., 2012; Salim et al., 2025).

Despite the recognized importance of ticks as disease vectors in Sudan, comprehensive information on their diversity and distribution remains limited. Available data are fragmented, often derived from scattered reports, outdated surveys, or studies restricted to specific regions or disease outbreaks (El Hussein et al., 2004; ElGhali and Hassan, 2012; Hassan and Salih, 2013). Early entomological investigations, some dating back to the mid-20th century, provided baseline descriptions of major tick species in Sudan (Hoogstraal, 1954, 1956). However, more recent studies remain limited and are frequently embedded within broader veterinary or epidemiological research, with insufficient updates to reflect the country's evolving ecological and climatic conditions (Mekki Osman, 1976; Latif, 1987; Salih et al., 2004; Hassan and Diaeldin, 2008).

Furthermore, political instability, prolonged conflict, and resource constraints have hindered the establishment and sustainability of effective entomological surveillance systems (Ahmed et al., 2020a, 2021; Mohamed et al., 2025). Consequently, the current understanding of tick species distribution and associated TBDs in Sudan is incomplete and potentially outdated. The lack of nationwide, standardized data limits the design and implementation of cost-effective control interventions (Lippi et al., 2021).

In addition, ongoing environmental and anthropogenic changes—including climate change, unplanned urbanization, globalization, increased travel and trade, and cross-border livestock movements—are driving the geographic expansion of tick populations, altering species composition, and influencing seasonal dynamics (Ahmed et al., 2021; Abubakr et al., 2025; Cao et al., 2025). In the absence of updated baseline data, disease control programs face significant challenges in developing and implementing effective preparedness, prevention, and response strategies (Estrada-Peña et al., 2012; Mansfield et al., 2017; Choi and Lee, 2025).

This review aims to provide a comprehensive synthesis of the diversity and distribution of ticks and TBDs in Sudan, identify critical knowledge gaps in vector ecology and disease control, and highlight priorities for future research and surveillance. Ultimately, this work seeks to inform policymakers, veterinary and public health stakeholders, and support evidence-based strategic planning for the development and implementation of sustainable and cost-effective control measures.

## 2 Review methodology

A comprehensive literature review was conducted to identify publicly available records and extract data on ticks and TBDs reported in Sudan. Systematic searches were performed across multiple online databases, including PubMed (https://pubmed.ncbi.nlm.nih.gov/), Web of Science (https://clarivate.com/), and CAB Abstracts (https://www.cabidigitallibrary.org/). Gray literature was systematically accessed through multiple sources, including university repositories (thesis and dissertations) and reports from national and international organizations including the World Health Organization (WHO), Food and Agriculture Organization (FAO), the World Organization for Animal Health (WOAH), and Sudanese Ministry of Health publications, as well as institutional databases and conference proceedings. Additional relevant studies were identified through screening the reference lists of selected articles. The search strategy combined controlled vocabulary and free-text terms using Boolean operators, including but not limited to: “ticks” OR “Ixodidae” OR “Argasidae” AND “tick-borne diseases” OR “TBDs” AND “Sudan.” Additional search terms included specific pathogen names (e.g., Crimean-Congo hemorrhagic fever, *Rickettsia, Babesia, Theileria*) and vector species commonly reported in Sudan.

The literature search was conducted between June and November 2025, and included studies published prior to 2026, to capture both historical baseline data and contemporary epidemiological evidence. No strict language restrictions were applied; however, where relevant, non-English records with English abstracts were screened for eligibility. Inclusion criteria were restricted to studies explicitly reporting on ticks and/or TBDs in Sudan. A detailed assessment of all identified records was conducted to ensure accurate geographic attribution and to avoid misclassification between Sudan and South Sudan, which became independent in 2011. All datasets were consolidated into a comprehensive inventory, with species names verified at the species level and duplicate records removed.

To ensure taxonomic consistency, species names were updated according to current classifications based on the systematic nomenclature proposed by Barker and Murrell (2004), while acknowledging that earlier classifications relied primarily on morphological descriptions following Hoogstraal (1956). The compiled dataset was subsequently used to assess patterns in species occurrence, distribution, and associated TBDs across Sudan.

## 3 Overview on ticks species

Ticks are obligate hematophagous ectoparasites that require the blood of vertebrate hosts, including humans and animals, for survival. Their host range is broad and includes mammals, reptiles, and birds. Globally, approximately 896 tick species have been described, with hard ticks (Ixodidae) representing the most diverse group, comprising around 702 recognized species. Many of these species play important roles as vectors of a wide range of pathogens affecting both humans and animals (Guglielmone et al., 2010).

Ticks are widely distributed across temperate, tropical, and subtropical regions, with species diversity strongly influenced by ecological factors, host availability, and climatic conditions. Their veterinary and public health importance arises from both direct and indirect effects. Direct effects include blood loss, skin irritation, and reduced productivity in livestock, while indirect effects result from the transmission of viral, bacterial, and protozoan pathogens that cause significant morbidity and mortality in animals and humans (Godfrey and Randolph, 2011).

Historically, ticks were classified into 19 genera; however, advances in molecular and genomic taxonomy have led to substantial revisions. For example, the former genera *Boophilus* and *Aponomma* are now considered subgenera within *Rhipicephalus* and *Amblyomma*, respectively. Currently, 18 genera are recognized worldwide, including *Argas, Ornithodoros, Amblyomma, Haemaphysalis, Rhipicephalus, Hyalomma, Ixodes, Rhipicentor, Nosomma, Margaropus, Dermacentor, Cornupalpatum, Cosmiomma, Bothriocroton, Anomalohimalaya, Otobius, Nuttalliella*, and *Carios* (Guglielmone et al., 2010).

### 3.1 Diversity and early documentation of tick species in Sudan before 2010

Historical records of tick distribution in Sudan date back to the Anglo-Egyptian Sudan period, prior to independence in 1956, when the country was divided into nine provinces: Northern, Kassala, Khartoum, Darfur, Kordofan, Blue Nile, Bahr El Ghazal, Upper Nile, and Equatoria. Following the separation of Sudan into two independent countries in 2011, the provinces of Bahr El Ghazal, Upper Nile, and Equatoria became part of South Sudan. Early surveys, particularly those by Hoogstraal between 1948 and 1952, documented a wide range of tick species within the present-day boundaries of Sudan (excluding South Sudan). These included *Argas brumpti, A. confusus, A. persicus, A. vespertilionis, Ornithodoros savignyi, Amblyomma lepidum, A. nuttalli, A. variegatum, Aponomma exornatum* (now classified within *Amblyomma* genus), *Boophilus annulatus* (currently *Rhipicephalus* (B.) *annulatus*), *B. decoloratus* (currently *R*. (B.) *decoloratus), Haemaphysalis houyi, H. leachii leachii, H. leachii muhsamae, Hyalomma excavatum* (now *H. anatolicum*), *H. detritum, H. dromedarii, H. impeltatum, H. impressum, H. marginatum, H. rufipes, H. truncatum, R. compositus, R. evertsi evertsi, R. sanguineus sanguineus*, and *R. simus simus* (Hoogstraal, 1954, 1956).

**Figure 1:**
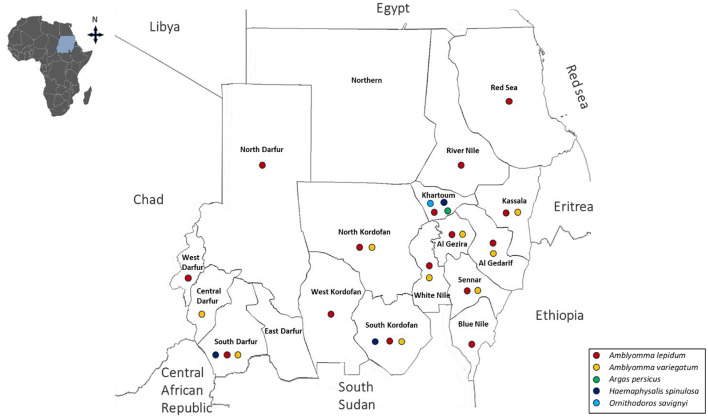
Historical distribution of *Amblyomma, Argas, Haemaphysalis*, and *Ornithodros* species in Sudan and South Sudan (formerly a single country of Sudan) up to 2010.

**Figure 2:**
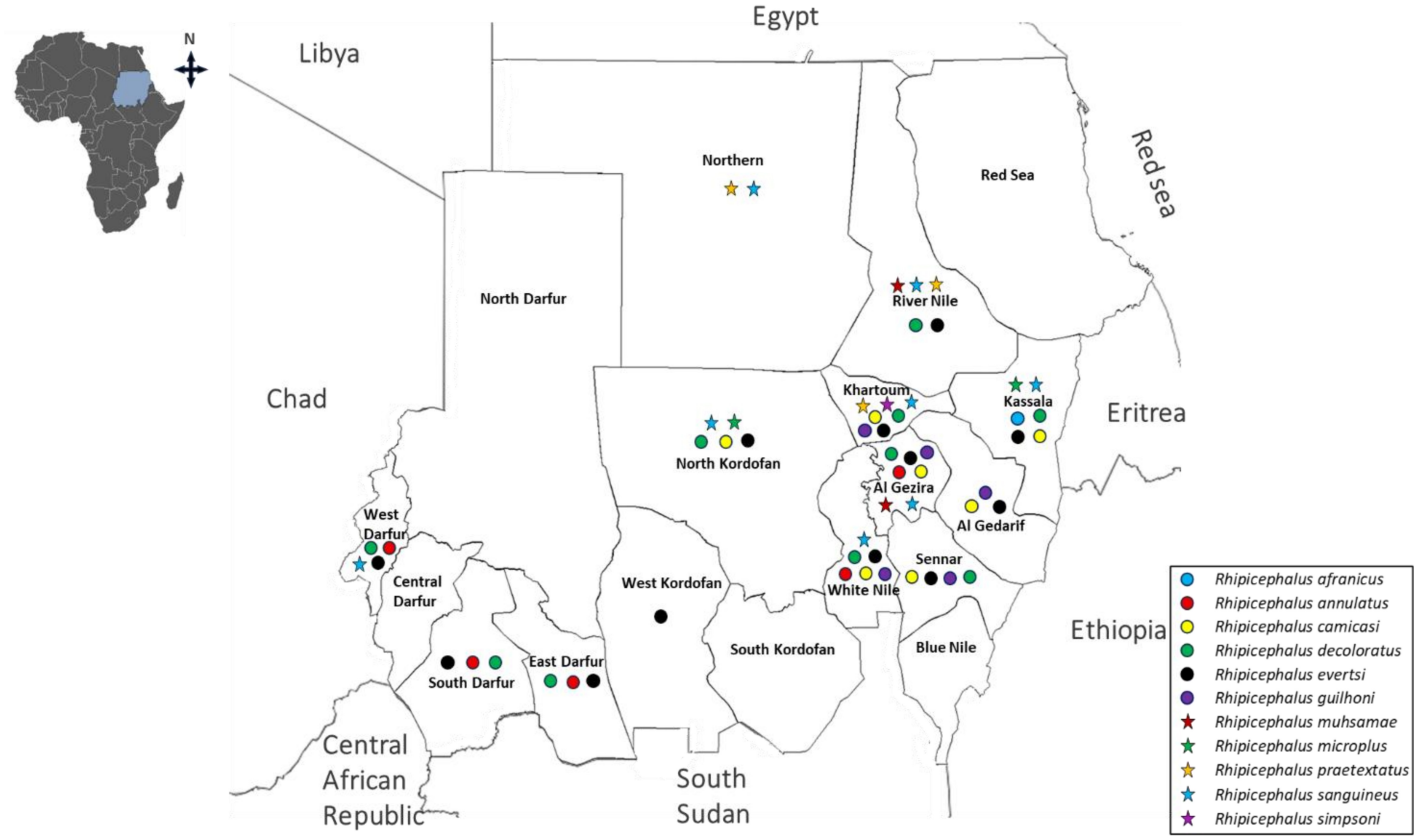
Historical distribution of *Hyalomma* species in Sudan and South Sudan (formerly a single country of Sudan) up to 2010.

**Figure 3:**
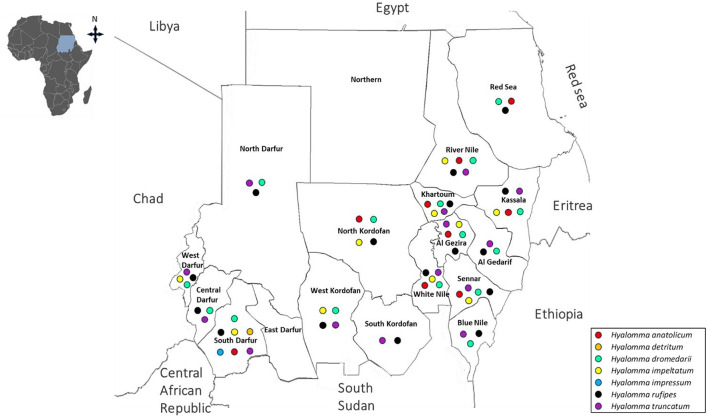
Historical distribution of *Rhipicephalus* species in Sudan and South Sudan (formerly a single country of Sudan) up to 2010.

Following independence in 1956 and prior to the 2010 division, multiple surveys were conducted across Sudan. However, these studies were often geographically limited and/or seasonally restricted, resulting in fragmented evidence of tick distribution. Collectively, these surveys documented 24 species across four major genera currently recognized in Sudan: *Amblyomma, Hyalomma, Rhipicephalus*, and *Haemaphysalis* species. Within the genus *Amblyomma, A. lepidum* and *A. variegatum* were the most widely distributed species, reported in 15 and 9 states, respectively (Karrar et al., 1963; Latif, 1984, 1985; Jongejan et al., 1987; Osman, 1997; Al Faki, 2004; Morita et al., 2004; Salih et al., 2004; Muramatsu et al., 2005; Mohammed and Hassan, 2007; Suliman, 2011; Alkareem et al., 2012; Gaafar et al., 2015; Nakao et al., 2015; Mohammed-Ahmed et al., 2018; Azrag et al., 2021). *Hyalomma* species exhibited both high diversity and widespread distribution. *H. rufipes* was recorded in 16 states, *H. dromedarii* in 15, *H. truncatum* in 14 states. *H. anatolicum* and *H. impeltatum* were also widely distributed, reported in 9 and 10 states, respectively. In contrast, species such as *H. detritum, H. impressum, H. marginatum*, and *H. turanicum* were more geographically restricted, each reported from only a single state (Karrar et al., 1963; Walker et al., 1983; Latif, 1984, 1985; Jongejan et al., 1987; Al Faki, 2004; Morita et al., 2004; Salih et al., 2004, 2005; Ahmed et al., 2005; Mohammed and Hassan, 2007; Elghali and Hassan, 2009; El Tigani and Mohammed, 2010; Taha et al., 2011; Alkareem et al., 2012; Gaafar et al., 2015; Yagoub et al., 2015; Elhaj et al., 2016; Mohammed-Ahmed et al., 2018). Similarly, *Rhipicephalus* species was also widely represented. *R. evertsi* was reported in 16 states, *R. sanguineus* in 13, and *R. decoloratus* in 12 states. Others species, including *R. camicasi* (10 states), *R. annulatus* and *R. guilhoni* (7 states each), and *R. simus* (8 states), showed moderate distribution. Less frequently reported species, such as *R. bergeoni, R. muhsamae, R. turanicus, R. geigyi*, and *R. sulcatus*, were confined to fewer states, while *R. senegalensis, R. caspidatus*, and *R. praetextatus* were recorded only once (Karrar et al., 1963; Latif, 1984, 1985; Hussein and Mustafa, 1985; Jongejan et al., 1987; Al Faki, 2004; Salih et al., 2004; Ahmed et al., 2005; Mohammed and Hassan, 2007; Taha et al., 2011; Alkareem et al., 2012; Gaafar et al., 2015; Yagoub et al., 2015; Elhaj et al., 2016; Mohammed-Ahmed et al., 2018). Less frequently reported genera included *Haemaphysalis*, with *H. spinulosa* identified in three states (Hussein and Mustafa, 1985). In contrast, soft ticks such as *A. persicus* and *O. savignyi* were each recorded in only a single state (Karrar et al., 1963; Osman, 1980) (Figures 1–3, Table 1).

**Table 1:** Early documentation of tick species in Sudan before 2010.

Tick genera	Species	States where ticks have been reported
*Amblyomma*	*A. lepidum*	Khartoum, Al Gezira, Sennar, White Nile, Al Gedarif, Kassala, River Nile, North Kordofan, South Kordofan, West Kordofan, South Darfur, North Darfur, West Darfur, Red Sea, and Blue Nile.
	*A. variegatum*	White Nile, Kassala, North Kordofan, South Kordofan, and South Darfur.
*Argas*	*A. persicus*	Khartoum, Al Gezira, Sennar, White Nile, Al Gedarif, Kassala, River Nile, North Kordofan, South Kordofan, West Kordofan, South Darfur, North Darfur, East Darfur, Central Darfur, West Darfur, Red Sea, Northern, and Blue Nile.
*Haemaphysalis*	*H. spinulosa*	Khartoum.
*Hyalomma*	*H. anatolicum*	Khartoum, Al Gezir, Sennar, White Nile, Kassala, River Nile, North Kordofan, South Darfur, and Red Sea.
	*H. detritum*	South Darfur
	*H. dromedarii*	Khartoum, Al Gezira, Sennar, White Nile, Al Gedarif, Kassala, River Nile, North Kordofan, West Kordofan, South Darfur, North Darfur, West Darfur, Red Sea, and Blue Nile.
	*H. excavatum*	Kassala, West Kordofan, and South Darfur.
	*H. impeltatum*	Khartoum, Al Gezira, Sennar, White Nile, Kassala, River Nile, North Kordofan, South Darfur, and West Darfur.
	*H. impressum*	South Darfur.
	*H. marginatum*	Kassala.
	*H. rufipes*	Khartoum, Al Gezira, Sennar, White Nile, Al Gedarif, Kassala, River Nile, West Kordofan, South Darfur, North Darfur, West Darfur, Red Sea, and Blue Nile.
	*H. truncatum*	Khartoum, Al Gezira, Sennar, White Nile, Al Gedarif, Kassala, West Kordofan, South Darfur, North Darfur, West Darfur, and Blue Nile.
	*H. turanicum*	Kassala.
*Rhipicephalus*	*R. annulatus*	Khartoum, Al Gezira, River Nile, South Darfur, and Blue Nile.
	*R. bergeoni*	Blue Nile.
	*R. camicasi*	Khartoum, Al Gezira, Sennar, White Nile, Al Gedarif, Kassala, River Nile, North Kordofan, South Darfur, and Blue Nile.
	*R. decoloratus*	Khartoum, Al Gezira, Sennar, White Nile, Kassala, River Nile, North Kordofan, West Kordofan, South Darfur, and Blue Nile.
	*R. evertsi*	Khartoum, Al Gezira, Sennar, White Nile, Al Gedarif, Kassala, River Nile, North Kordofan, West Kordofan, South Darfur, North Darfur, West Darfur, Red Sea, and Blue Nile.
	*R. guilhoni*	Al Gezira, Sennar, White Nile, Al Gedarif, North Kordofan, South Darfur, and Blue Nile.
	*R. muhsamae*	Khartoum, Sennar, and White Nile.
	*R. sanguineus*	Khartoum, Al Gezira, Sennar, White Nile, Al Gedarif, Kassala, River Nile, West Kordofan, South Darfur, North Darfur, West Darfur, and Blue Nile.
	*R. senegalensis*	Khartoum.
	*R. simus*	Khartoum, White Nile, Kassala, River Nile, West Kordofan, and Blue Nile.
	*R. turanicus*	Al Gezira, Sennar, White Nile, and Blue Nile.

### 3.2 Distributions of tick species in the current Sudan (post-2010)

Tick surveillance in Sudan after 2010 has been characterized by fragmented yet valuable efforts, with most records derived from regional surveys or complementary investigations conducted in the context of veterinary research. These studies have primarily focused on documenting the presence and diversity of tick species across different states, often in relation to their role as vectors of economically important livestock diseases. Rather than through nationwide systematic mapping, most post-2010 data have emerged as byproducts of targeted animal health research, which nevertheless provide important insights into the persistence and expansion of tick populations across the country. Findings from these studies confirm the continued presence of previously reported genera, including *Amblyomma, Hyalomma*, and *Rhipicephalus*. However, several reports also indicate shifts in their geographical distribution patterns, likely driven by changes in agro-pastoral practices, livestock movement, land use and land cover, and ecological dynamics associated with climate variability.

**Figure 4:**
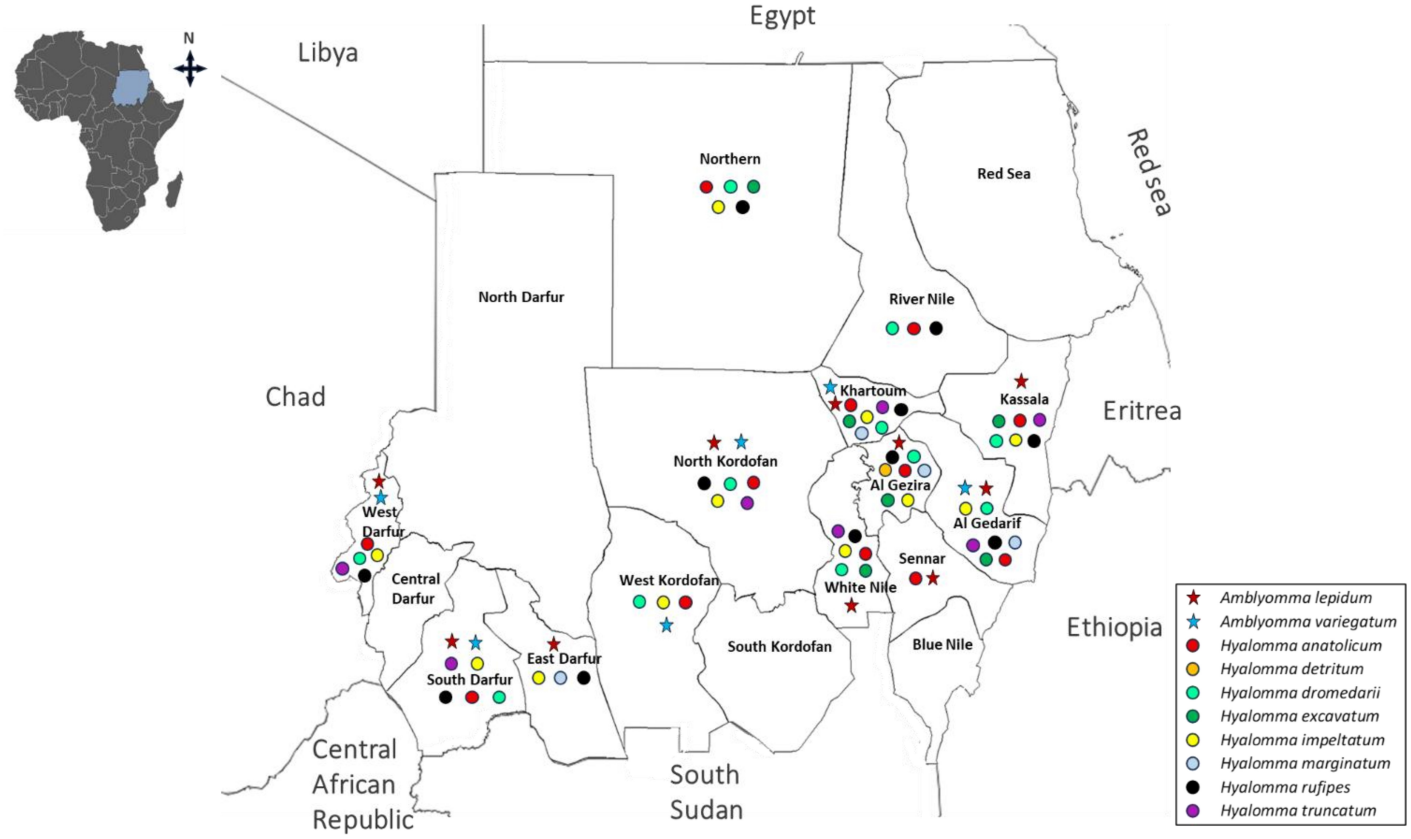
Distributions of *Amblyomma* and *Hyalomma* species in the current Sudan (post-2010).

**Figure 5:**
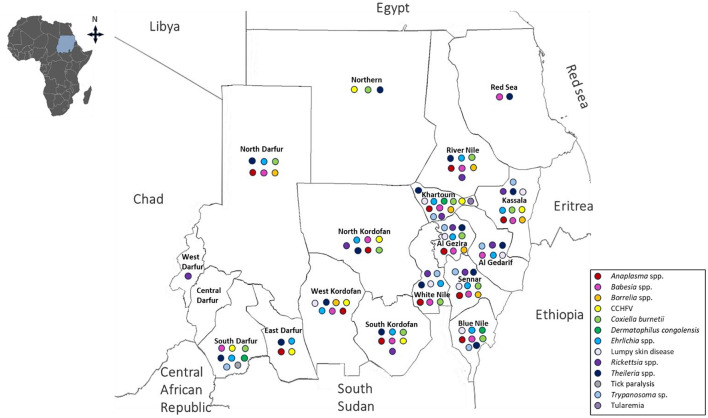
Distributions of *Rhipicephalus* species in the current Sudan (post-2010).

Within the genus *Amblyomma, A. lepidum* remains relatively widespread, reported in 10 states, whereas *A. variegatum* shows a more restricted distribution, occurring in five states (Ali, 2012; Guma et al., 2015; Abaker et al., 2017; Bala et al., 2018a,b; Mohammed et al., 2018b; Hayati et al., 2020; Shuaib et al., 2020a,b; Mossaad et al., 2021; Eisawi et al., 2024). *Hyalomma* species continue to dominate the tick fauna. *H. anatolicum, H. dromedarii*, and *H. impeltatum* have each been reported in approximately 12 states. Other species, including *H. rufipes* (11 states) and *H. truncatum* (7 states), also show broad distribution, whereas *H. marginatum* (4 states) appears more localized, and *H. detritum* remains rare, reported in only a single state (Ali, 2012; Guma et al., 2015; Abaker et al., 2017; Bala et al., 2018a,b; Hassan et al., 2018; Mohammed et al., 2018b, 2022; Chitimia-Dobler et al., 2019; Elhaj et al., 2019; Hayati et al., 2020; Shuaib et al., 2020a,b; Magzoub et al., 2021; Mossaad et al., 2021; Eisawi et al., 2024). *Rhipicephalus* species display both widespread and more restricted distributions. *R. evertsi* which was the most widely reported species (12 states), followed by *R. decoloratus* (10 states), *R. sanguineus* (8 states), and *R. camicasi* (7 states). Moderate distribution is observed for *R. annulatus* and *R. guilhoni* (5 states each), while less frequently reported species include *R. praetextatus* (3 states), *R. microplus* and *R. muhsamae* (2 states each), and *R. turanicus* (reported as *R. afranicus*) and *R. simpsoni*, both restricted to a single state (Ali, 2012; Guma et al., 2015; Abaker et al., 2017; Bala et al., 2018a,b; Hassan et al., 2018; Mohammed et al., 2018b, 2022; Elhaj et al., 2019; Hayati et al., 2020; Shuaib et al., 2020a,b; Magzoub et al., 2021; Mossaad et al., 2021; Eisawi et al., 2024) (Figures 4, 5, Table 2). Notably, some studies have reported the emergence of species not previously documented in Sudan, such as *R. microplus* in Kassala State. This finding aligns with earlier predictions by Hoogstraal, who suggested that this species could potentially establish in Sudanese ecosystems (Hoogstraal, 1956). Despite these contributions, important gaps remain in post-2010 surveillance, as several regions—including Red Sea, Central Darfur, North Darfur, South Kordofan, and Blue Nile states—lack recent entomological data.

**Table 2:** Distributions of tick species in the current Sudan (post-2010).

Tick genera	Species	States where ticks have been reported
*Amblyomma*	*A. lepidum*	Khartoum, Al Gezira, Sennar, White Nile, Al Gedarif, Kassala, North Kordofan, South Darfur, East Darfur, and West Darfur.
	*A. variegatum*	Khartoum, Al Gezira, Sennar, White Nile, Al Gedarif, North Kordofan, South Kordofan, South Darfur, and West Darfur.
*Hyalomma*	*H. anatolicum*	Khartoum, Al Gezira, Sennar, White Nile, Al Gedarif, Kassala, River Nile, North Kordofan, West Kordofan, South Darfur, West Darfur, and Northern.
	*H. detritum*	Al Gezira.
	*H. dromedarii*	Khartoum, Al Gezira, White Nile, Al Gedarif, Kassala, River Nile, North Kordofan, West Kordofan, South Darfur, East Darfur, West Darfur, and Northern.
	*H. excavatum*	Khartoum, Al Gezira, Sennar, White Nile, Al Gedarif, Kassala, and Northern.
	*H. impeltatum*	Khartoum, Al Gezira, White Nile, Al Gedarif, Kassala, North Kordofan, West Kordofan, South Darfur, West Darfur, and Northern.
	*H. marginatum*	Khartoum, Al Gezira, Al Gedarif, and East Darfur.
	*H. rufipes*	Khartoum, Al Gezira, Sennar, White Nile, Al Gedarif, Kassala, River Nile, North Kordofan, South Darfur, East Darfur, West Darfur, and Northern.
	*H. truncatum*	Khartoum, White Nile, Al Gedarif, Kassala, North Kordofan, South Darfur, and West Darfur.
*Rhipicephalus*	*R. afranicus*	Kassala.
	*R. annulatus*	Al Gezira, White Nile, South Darfur, East Darfur, and West Darfur.
	*R. camicasi*	Khartoum, Al Gezira, Sennar, White Nile, Al Gedarif, Kassala, and North Kordofan.
	*R. decoloratus*	Khartoum, Al Gezira, Sennar, White Nile, Kassala, River Nile, North Kordofan, South Darfur, East Darfur, and West Darfur.
	*R. evertsi*	Khartoum, Al Gezira, Sennar, White Nile, Al Gedarif, Kassala, River Nile, North Kordofan, West Kordofan, South Darfur, East Darfur, and West Darfur.
	*R. guilhoni*	Khartoum, Al Gezira, Sennar, White Nile, and Al Gedarif.
	*R. microplus*	Kassala and North Kordofan.
	*R. muhsamae*	Al Gezira and River Nile.
	*R. praetextatus*	Khartoum, River Nile, and Northern.
	*R. sanguineus*	Khartoum, Al Gezira, White Nile, Kassala, River Nile, North Kordofan, West Darfur, and Northern.
	*R. simpsoni*	Khartoum.

Comparisons of tick records before and after 2010 reveal both continuity and notable shifts in distribution patterns. Some species, such as *H. anatolicum, H. marginatum*, and *R. praetextatus*, appear to have expanded their geographic ranges, whereas *R. microplus* and *R. simpsoni* have only been reported in the post-2010 period. In contrast, species previously considered widespread—such as *A. lepidum, H. rufipes*, and *R. evertsi*—showed reduced reporting frequencies, while others, including *R. simus, H. turanicum*, and *H. spinulosa*, are absent from post-2010 records. These apparent changes should not be interpreted solely as true ecological expansions or contractions. Rather, they likely reflect the fragmented and localized nature of tick surveillance in Sudan, where variations in study design, geographic coverage, and sampling methodologies influence species detection and reporting. This highlights a major gap in coordinated national medical and veterinary entomological surveillance systems. For example, reports on *Argas* and *Ornithodoros* species remain scarce in both pre- and post-2010 periods, despite evidence that soft ticks such as *Argas persicus* continue to impose significant economic burdens on poultry production (Hoogstraal, 1956; Zahid et al., 2023).

## 4 Overview on TBDs

TBDs represent major public health and veterinary concerns in Sudan due to their substantial impacts on health, socioeconomic stability, and economic growth. Ticks act as vectors of a wide range of pathogens affecting both humans and animals (Ahmed et al., 2025a,c).

In animals, some of the most important TBDs include theileriosis, babesiosis, anaplasmosis, and ehrlichiosis, all of which contribute to significant economic losses through reduced productivity, increased mortality, and the high costs of treatment and control measures (El Hussein et al., 2004). Among these, tropical theileriosis and East Coast fever—caused by *T. annulata* and *T. parva*, respectively, and transmitted by *Hyalomma* and *Rhipicephalus* ticks—pose major threats to cattle health (Gharbi et al., 2020).

In poultry, avian spirochetosis, caused by *Borrelia anserina*, is the most recognized tick-associated disease (Ouchene et al., 2020). In Sudan, outbreaks of avian spirochetosis were reported in Khartoum State between 2003 and 2007. Although no recent outbreaks have been documented, the disease continues to be reported in commercial layer farms (Hamza, 2008; El Nasri et al., 2010). This apparent scarcity of recent reports likely reflects limited surveillance in poultry systems compared with other livestock sectors, as well as the predominant focus of tick research in Sudan on ruminants with higher economic and export value (Ahmed, 2020; Ahmed et al., 2022). This underrepresentation highlights how gaps in research priorities can obscure the contribution of less-studied vectors and hosts, despite their substantial impact on local production systems.

In humans, the most frequently reported TBD in Sudan is Crimean–Congo hemorrhagic fever (CCHF), with recurrent outbreaks occurring across the country. These outbreaks often arise in endemic regions and may involve healthcare settings, posing significant risks to healthcare workers (Aradaib et al., 2010; Ahmed et al., 2022). Other TBDs reported in Sudan include relapsing fever and spotted fever group rickettsioses, which are associated with considerable morbidity and, in some cases, high mortality (Cutler, 2015; Ahmed et al., 2022; Zhang et al., 2023).

The risk of transmission is amplified by close interactions between humans, livestock, and ticks in pastoral and agro-pastoral communities, where animal husbandry practices facilitate both tick proliferation and human exposure. In addition, climatic variability, cross-border livestock movement, and limited surveillance capacity contribute to the spread and underreporting of these diseases (Murray, 2024).

### 4.1 Global diversity of viral TBDs

Ticks serve as important vectors of a diverse range of arboviruses, including both RNA and DNA viruses, affecting humans, livestock, and wildlife. The severe impact of tick-borne viruses is underscored by the fact that several of these agents cause hemorrhagic fevers, some of which are fatal, as well as neurological diseases in human and animals, and economically significant losses in livestock (Labuda and Nuttall, 2004; Shi et al., 2018).

Among these viruses, African swine fever virus (family Asfarviridae) is particularly devastating, causing acute hemorrhagic disease in pigs with high mortality rates and posing major challenges to animal health, global health, and food security (Sanchez-Cordon et al., 2018). Similarly, members of the order *Bunyavirales*, particularly nairoviruses such as Crimean–Congo hemorrhagic fever virus (CCHFV), are of major public health importance due to their ability to cause severe hemorrhagic fever with high case-fatality rates. Other bunyaviruses, including Dugbe, Dhori, Bhanja, Sawgrass, Nairobi sheep disease virus, and Thogoto virus, are associated with febrile illness in humans and livestock, and in some cases may progress to neurological or hemorrhagic disease manifestations (Ahmed et al., 2025a).

Other tick-associated viruses of veterinary importance include lumpy skin disease virus (family Poxviridae, genus *Capripoxvirus*), which causes characteristic cutaneous nodules in cattle and results in substantial economic losses in endemic regions (Sprygin et al., 2019). In addition, bluetongue virus (family Reoviridae, genus *Orbivirus*), although primarily transmitted by *Culicoides* midges, has been discussed in the context of potential tick involvement and causes hemorrhagic disease and significant morbidity in ruminants (Bouwknegt et al., 2010).

Recently emerging tick-associated phleboviruses in North America, including Heartland virus and Bourbon virus, as well as severe fever with thrombocytopenia syndrome virus (SFTSV) in East Asia, highlight the expanding geographic distribution and zoonotic potential of tick-borne phleboviruses. Heartland virus was first identified in two severely ill farmers in Missouri and has since been associated with human cases characterized by fever, leukopenia, and thrombocytopenia, with evidence implicating *A. americanum* (Lone Star tick) as a vector (McMullan et al., 2012). Bourbon virus was identified in 2014 following a fatal human case in Kansas (Kosoy et al., 2015). Although its sylvatic cycle and primary vector remain incompletely defined, epidemiological associations with tick exposure, along with viral detections in ticks and wildlife, support a zoonotic transmission cycle (Savage et al., 2017; Dupuis et al., 2023; Roe et al., 2023).

SFTSV, first described in China in 2010, has been associated with large outbreaks in East Asia and exhibiting higher case-fatality rates than many other arboviral febrile illnesses (Zheng et al., 2025). The virus is transmitted by *H. longicornis*, which also serves as a competent vector and potential reservoir, highlighting its epidemiological importance (Luo et al., 2015). Its emergence underscores how novel phleboviruses can cross the human–animal interface with significant public health consequences (Liu et al., 2014).

Other tick-borne virus families include Reoviridae, represented by Colorado tick fever virus, transmitted by *D. andersoni*, which causes a biphasic febrile illness in humans (Fagre et al., 2024). Members of the family Flaviviridae are also of major importance due to their neurotropic and hemorrhagic potential. These include tick-borne encephalitis virus transmitted by *I. ricinus* in Europe and *I. persulcatus* in Asia; Powassan virus in North America transmitted by *I. scapularis, I. cookei*, and *I. marxi*; Kyasanur Forest disease virus in India transmitted by *H. spinigera*; Omsk hemorrhagic fever virus in Russia transmitted by *D. reticulatus*; Alkhumra virus in Egypt transmitted by *O. savignyi* and *H. dromedarii*; and Louping ill virus in sheep transmitted by *I. ricinus* (Calisher, 1988; Hudson et al., 1995; Amicizia et al., 2013; Musso et al., 2015; Robich et al., 2019; Diani et al., 2025; Pandey and Singh, 2025).

### 4.2 Global diversity of parasitic TBDs

Ticks are recognized as vectors of several protozoan parasites with substantial veterinary and, in some cases, human health importance (Ahmed et al., 2025c). Among the less commonly reported parasites is *Anthemosoma garnhami*, originally described in rodents but subsequently detected in other mammals, including humans, where it can cause a malaria-like illness (Landau et al., 1969; Stead et al., 2021).

More widely recognized is babesiosis (piroplasmosis), caused by diverse *Babesia* species, which represents an important zoonotic disease. In humans, infection may present with malaria-like symptoms, while in cattle it leads to severe anemia, fever, and substantial production losses (Dantas-Torres et al., 2017). Cytauxzoonosis, caused by *Cytauxzoon felis*, is another tick-borne protozoan infection that is typically fatal in domestic cats. Transmission is usually associated with *A. americanum* or *D. variabilis*, facilitating spillover from wildlife reservoirs, particularly wild felids such as lynx (Cohn and Birkenheuer, 2021).

In livestock, theileriosis caused by *Theileria* species is among the most important tick-borne parasitic diseases in Africa and Asia. It induces lymphoproliferative disorders that severely compromise cattle health and productivity (Mukhebi et al., 1992; Yin et al., 2007; Ahmed et al., 2025c). Other protozoan pathogens include *Hepatozoon* species, which cause hepatozoonosis in dogs and a range of vertebrates (Giannelli et al., 2017). Unlike most tick-borne pathogens, transmission occurs through ingestion of infected ticks (e.g., *R. sanguineus* or *A. maculatum*) rather than via tick bite (Smith, 1996).

Although less frequently implicated, ticks have also been associated with the transmission of certain *Trypanosoma* species affecting animals (Kernif et al., 2024). Similarly, while *Leishmania* species are primarily transmitted by sandflies, occasional reports suggest that ticks may play a secondary or mechanical role in transmission dynamics (Kernif et al., 2024).

### 4.3 Global diversity of bacterial TBDs

Among bacterial pathogens, rickettsial and ehrlichial infections represent the most prominent tick-borne bacterial diseases. *Rickettsia africae*, the causative agent of African tick-bite fever, is widely distributed in sub-Saharan Africa and causes an acute febrile illness often accompanied by eschar and rash, particularly in travelers (Silva-Ramos and Faccini-Martínez, 2021). Within the spotted fever group, *R. conorii* (Mediterranean spotted fever) and *R. rickettsii* (Rocky Mountain spotted fever) are among the most severe and potentially fatal systemic infections (Silva-Ramos and Faccini-Martínez, 2021).

Closely related pathogens belong to the family *Anaplasmataceae*. *Anaplasma phagocytophilum* causes human granulocytic anaplasmosis by infecting neutrophils (Matei et al., 2019), while *Ehrlichia chaffeensis* is responsible for human monocytic ehrlichiosis. Both infections are characterized by fever, cytopenias, and potentially life-threatening complications (Brouqui et al., 1994). Newly identified organisms such as Panola Mountain Ehrlichia further expand the known diversity of tick-borne *Ehrlichia* species (Reeves et al., 2008).

Other bacterial agents further highlight the broad spectrum of TBDs. Lyme disease, caused by spirochetes of the *Borrelia burgdorferi* sensu lato complex, represents the most common tick-borne bacterial infection in temperate regions. It is transmitted primarily by *Ixodes* species and is characterized by multisystem involvement, ranging from erythema migrans to arthritis and neuroborreliosis (Rudenko et al., 2011; Ivanova et al., 2014; Pritt et al., 2016).

Q fever, caused by *Coxiella burnetii*, is a zoonotic infection that may present as an acute febrile illness or progress to chronic disease, including endocarditis (Melenotte et al., 2018). The pathogen is maintained primarily in livestock reservoirs, with ticks contributing to its transmission among animal hosts (Celina and Cerný, 2022). Tularemia, caused by *Francisella tularensis*, can be transmitted through tick bites and may result in ulceroglandular disease with potential systemic dissemination (Petersen et al., 2009; Genchi et al., 2015). Additionally, emerging evidence suggests that *Bartonella henselae*, the agent of cat-scratch disease, may also be transmitted by ticks; however, its epidemiological role in tick-borne transmission remains under investigation (Mosbacher et al., 2010).

### 4.4 Role of ticks in the transmission and transportation of fungal pathogens

Despite the scarcity of studies on tick-borne fungal pathogens, accumulating evidence suggests that several tick species may be involved in the acquisition, carriage, and potential transmission of diverse fungal taxa. A recent study investigating the fungal microbiome of Ixodidae ticks in China reported the presence of 261 fungal genera across four taxonomic classes in *D. nuttalli* ticks (Sun et al., 2024). Several of these genera include species of medical and veterinary importance, with known human, animal, and zoonotic pathogenic potential.

### 4.5 Ticks-associated syndromes and conditions

Ticks are not only vectors of pathogens but are also directly responsible for a range of medical and veterinary conditions arising from their feeding behavior, salivary components, and associated skin trauma. One emerging example is alpha-gal syndrome, an immunoglobulin E–mediated hypersensitivity reaction to galactose-α-1,3-galactose, a carbohydrate introduced into humans through tick bites. This syndrome is characterized by delayed anaphylaxis or urticaria following the consumption of mammalian meat products, highlighting the expanding role of ticks in allergic diseases (Commins and Platts-Mills, 2013; Cabezas-Cruz et al., 2019).

Another well-recognized condition is tick paralysis, which results from neurotoxins secreted by engorging female ticks, particularly species of *Dermacentor* and *Ixodes*. These neurotoxins interfere with neuromuscular transmission, leading to ascending flaccid paralysis that may progress to respiratory failure and death if the tick is not removed promptly (Grattan-Smith et al., 1997; Mans et al., 2004).

In livestock, sweating sickness is a toxicosis associated with salivary secretions of certain *Hyalomma* tick species. Affected cattle present with fever, profuse sweating, and cutaneous lesions, often with high mortality, thereby imposing a significant burden on animal health and productivity in affected regions (Heyman et al., 2018).

Similarly, dermatophilosis, caused by *Dermatophilus congolensis*, is frequently associated with tick infestation and other skin-traumatizing factors. This exudative dermatitis affects cattle, sheep, goats, and occasionally humans, with clinical manifestations ranging from crusted lesions to widespread skin infection, particularly under conditions of high humidity and heavy ectoparasite burden (Zaria, 1993; Ambrose et al., 1999).

## 5 TBDs in Sudan

In Sudan, several TBDs have been reported, particularly among animals. Although human infections also represent a substantial proportion of the burden, diagnostic constraint remains a major limitation in the detection and confirmation of these infections in humans (Ahmed et al., 2025a).

**Figure 6:**
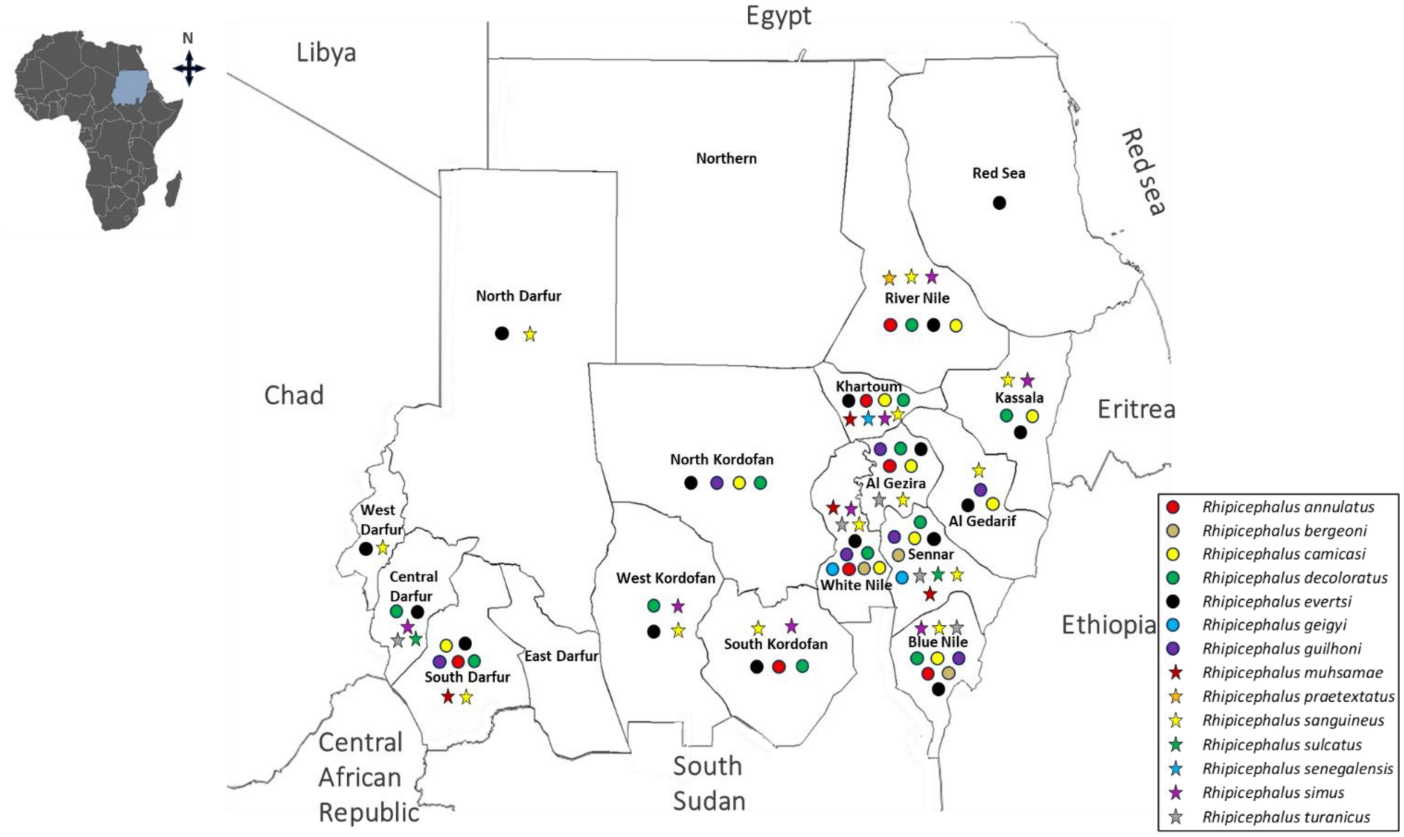
Geographical distribution of tick-borne diseases in Sudan.

Although records of TBDs in Sudan date back to the early 19th century, several regions of the country have historically lacked documented reports of disease occurrence. More recently, evidence has emerged indicating a substantial risk of transmission, supported by reports from neighboring countries and high levels of cross-border livestock movement. Figure 3 illustrates the diversity of TBDs and associated conditions reported in Sudan (Figure 6, Table 3).

**Table 3:** Reported tick-borne diseases, associated ticks, hosts, and state-level distribution in Sudan.

Disease	Ticks reported carrying the pathogen^*^	Infected host	States where disease reported
Anaplasmosis	*R. annulatus, R. decoloratus, R. evertsi R. praetextatus, H. rufipes, H. anatolicum, H. impeltatum, H. marginatum*	Cattle, Sheep, Horse, Goat, Dog	Al Gezira, Blue Nile, East Darfur, Kassala, Khartoum, North Darfur, North Kordofan, River Nile, Sennar, South Kordofan, West Kordofan, and White Nile
Babesiosis	*R. annulatus, R. decoloratus, R. evertsi, R. praetextatus, H. rufipes, H. anatolicum, H. impeltatum, H. marginatum, A. lepidum, A. variegatum*	Cattle, Sheep, Horse, Goat, Donkey, Camel	Al Gedarif, Al Gezira, Blue Nile, Kassala, Khartoum, Kassala, North Darfur, North Kordofan, Red Sea, River Nile, Sennar, South Darfur, South Kordofan, West Kordofan, White Nile
Borreliosis	Associated with *Ornithodoros* and *Ixodes* species	Chicken, Goat	River Nile, Kassala, North Darfur, West Kordofan, Al Gezira, Khartoum, Sennar
Crimean-Congo hemorrhagic fever	Associated with *Hyalomma* species	Cattle, Camel, Human	Northern, West Kordofan, East Darfur, North Kordofan, South Kordofan, Khartoum, South Darfur, Kassala
Q fever	Associated with *Hyalomma, Rhipicephalus*, and *Amblyomma* species	Cattle, Human, Goat, Camel	Al Gezira, Blue Nile, Kassala, Khartoum, North Kordofan, North Darfur, Northern, River Nile, Sennar, South Darfur, South Kordofan, White Nile
Ehrlichiosis	*R. sanguineus, R. evertsi, A. lepidum, H. anatolicum, A. variegatum*	Sheep, Goat, Camel, Cattle, Dog	Al Gedarif, Al Gezira, Blue Nile, East Darfur, Kassala, Khartoum, North Darfur, North Kordofan, River Nile, Sennar, South Darfur, South Kordofan, West Kordofan, and White Nile
Rickettsiosis	*H. rufipes, A. lepidum, A. variegatum, H. dromedarii, H. impeltatum, H. anatolicum, H. truncatum, R. evertsi, R. camicasi, R. decoloratus, R. afranicus, H. marginatum*	Cattle, Sheep Goat	Al Gedarif, Al Gezira, Kassala, Khartoum, North Kordofan, River Nile, Sennar, South Kordofan, West Darfur, and White Nile
Lumpy skin disease	No reported ticks	Cattle	Blue Nile, Al Gezira, Kassala, Al Gedarif, Khartoum, West Kordofan, White Nile, Sennar
Theileriosis	*R. evertsi, R. praetextatus, H. rufipes, H. anatolicum, H. impeltatum, H. marginatum, A. lepidum, R. camicasi*	Cattle, Sheep, Horse, Donkey, Goat, Gazelle	Al Gedarif, Al Gezira, Blue Nile, East Darfur, Kassala, Khartoum, North Darfur, North Kordofan, Northern, Red Sea, River Nile, Sennar, South Darfur, South Kordofan, West Kordofan, and White Nile
Tick paralysis	Neurotoxin from *Hyalomma* and *Rhipicephalus* species	Camel	South Darfur
Dermatophilosis	Associated with *Amblyomma* and *Hyalomma* species	Camel, Cattle	Khartoum, Al Gezira, South Darfur
Tularemia	Associated with *Dermacentor* and *Amblyomma* species	Human	Khartoum
Animal trypanosomiasis	*H. anatolicum*	Cattle, Horse, Camel	Sennar, Al Gezira, Khartoum, White Nile, South Darfur, Al Gedarif, Kassala, and Blue Nile

^*^Ticks reported associated with the pathogens were not reported in Sudan. Studies were serological investigations on hosts.

### 5.1 Anaplasmosis

Anaplasmosis is one of the most prevalent tick-borne infections in Sudan, particularly in cattle. It is caused by obligate intracellular bacteria of the genus *Anaplasma*, mainly *A. marginale* in cattle and *A. ovis* in sheep and goats (Lee et al., 2018; Mossaad et al., 2021). Transmission occurs primarily through *Rhipicephalus* and *Hyalomma* ticks, which are widespread in Sudan's livestock-producing regions (Bakheit et al., 2012). In addition to domestic animals, wildlife are believed to contribute to the natural maintenance cycle of the pathogen, with wild ungulates proposed as potential reservoir hosts (Kocan et al., 2010).

Clinical disease in cattle is characterized by fever, anemia, jaundice, and weight loss, with mortality increasing under stress conditions such as drought or co-infections. Small ruminants and camels are also susceptible, although infections are often subclinical (Dantas-Torres and Otranto, 2016). Economic losses arise from reduced milk production, poor growth rates, and reproductive inefficiencies (Railey and Marsh, 2021). Human anaplasmosis has not yet been well characterized in Sudan; however, serological surveillance is warranted to assess its zoonotic potential, given that human cases have been reported in regions such as Cyprus and the United States, often associated with tick exposure (Dumler et al., 2005; Chochlakis et al., 2010).

Anaplasmosis affecting horses, cattle, sheep, goats, and dogs has been widely reported across Sudan, with confirmed occurrences in Al Gezira (Latif, 1987; Awad et al., 2011), Blue Nile (Salih et al., 2009; Lee et al., 2018), East Darfur (Mossaad et al., 2021), Kassala (Inokuma et al., 2006; Salih et al., 2009), Khartoum (Latif, 1987; Ibrahim et al., 2010; Eisawi et al., 2020; Mossaad et al., 2021; Satti et al., 2021), North Darfur (Salih et al., 2009), North Kordofan (Salih et al., 2009; El Ayis, 2022), River Nile (Salih et al., 2009; Awad et al., 2011), Sennar (Latif, 1987; Salih et al., 2009; Mohammed et al., 2018a), South Kordofan (Sowar, 2002; Zein et al., 2013), West Kordofan (Lee et al., 2018; El Ayis, 2022), and White Nile states (Awad et al., 2011) (Figure 6).

### 5.2 Babesiosis

Babesiosis, commonly referred to as “redwater” in livestock, is caused by protozoan parasites such as *B. bovis* and *B. bigemina* in cattle and is transmitted primarily by *Rhipicephalus* ticks (Mossaad et al., 2021). In Sudan, bovine babesiosis is endemic and represents a significant veterinary challenge (Latif, 1987).

The disease is characterized by fever, anemia, hemoglobinuria, and, in severe acute cases, neurological complications. In sheep, *B. ovis* is the principal causative agent, particularly in arid pastoral systems (Firat et al., 2024). Babesiosis contributes substantially to economic losses in Sudan through livestock mortality, reduced milk and meat production, and increased costs associated with treatment and acaricide application. Control remains challenging due to acaricide resistance and the persistence of carrier animals, including cattle, sheep, and dogs (Oyamada et al., 2005; Salih et al., 2009).

Similar to anaplasmosis, human babesiosis has not been documented in Sudan; however, its occurrence cannot be excluded given close human–animal contact and the presence of competent vectors in several regions. Nevertheless, limited diagnostic capacity may hinder detection, as babesiosis can be easily misdiagnosed as other febrile illnesses such as malaria or typhoid fever (Krause, 2019).

In Sudan, babesiosis in domestic animals—including cattle, sheep, horses, donkeys, goats, and dogs—has been confirmed across multiple states, Al Gedarif (Salih et al., 2009; Ibrahim et al., 2017), Al Gezira (Latif, 1987; Saad, 2004; Salih et al., 2009; Awad et al., 2011; Abdelgalil et al., 2023), Blue Nile (Abdoon, 1984; Salih et al., 2009; Lee et al., 2018), Kassala (Salih et al., 2009; Awad et al., 2011; Springer et al., 2020), Khartoum (Abdalla, 1984; Abdoon, 1984; Latif, 1987; Abdoon et al., 1992; Ibrahim et al., 2010; Mossaad et al., 2021; Satti et al., 2021), North Darfur (Abdelrahim et al., 2009; Salih et al., 2009), North Kordofan (Salih et al., 2009; Springer et al., 2020; El Ayis, 2022), Red Sea (Salih et al., 2009), River Nile (Salih et al., 2009; Awad et al., 2011), Sennar (Latif, 1987; Salih et al., 2009; Mohammed et al., 2018a), South Darfur (Saad, 2004; Adam, 2005; Salih et al., 2009; Idris et al., 2018), South Kordofan (Sowar, 2002; Saad, 2004; Basheir et al., 2012), West Kordofan (Alkareem et al., 2012; El Ayis, 2022), and White Nile (Abdoon, 1984; Salih et al., 2009; Awad et al., 2011; Bala et al., 2018a) (Figure 6).

### 5.3 Borreliosis

Borreliosis, also known as tick-borne relapsing fever (TBRF), is caused by bacteria of the genus *Borrelia*, with *B. crocidurae* and *B. duttonii* among the principal species implicated in human disease (Parola et al., 2011). In Sudan, TBRF is characterized by recurrent febrile episodes, headache, and myalgia. Outbreaks have been reported and are often associated with poor housing conditions that facilitate infestation by *Ornithodoros* ticks. Children and displaced populations are particularly vulnerable (Salih et al., 1977; WHO, 1999). Despite its public health importance, borreliosis remains underdiagnosed due to symptom overlap with malaria and limited laboratory diagnostic capacity (Doss et al., 2024).

Borrelial infections among animals were reported from River Nile (El Hussein et al., 2004), Sennar (Suliman and Mamoun, 1997), Khartoum (Hassan and Diaeldin, 2008), Kassala, North Darfur, West Kordofan, and Al Gezira states (Kirk, 1940) (Figure 6). The observed distribution, with higher reporting in central and eastern states and more limited records in western regions, likely reflects the ecology of soft ticks (*Ornithodoros* spp.), which often remain undetected in conventional surveillance due to their cryptic behavior. These ticks inhabit concealed microenvironments such as animal burrows and nests and exhibit rapid feeding on specific hosts, including rodents, birds, bats, and livestock, rather than actively questing on vegetation (Hoogstraal, 1985). This pattern may also reflect differences in diagnostic attention and surveillance intensity, suggesting that underreporting in other pastoral regions is likely.

### 5.4 Crimean-Congo hemorrhagic fever

CCHFV is one of the most serious zoonotic tick-borne viral pathogens in Sudan (Ahmed et al., 2022). The virus is maintained in nature through *Hyalomma* ticks, which are widely distributed and commonly found in livestock herds across the country. Multiple outbreaks have been reported, with sporadic human cases, including infections among healthcare workers (Ahmed et al., 2022).

In humans, CCHF is characterized by sudden onset of fever, myalgia, dizziness, and gastrointestinal symptoms. In severe cases, the disease progresses to hemorrhagic manifestations with case fatality rates of up to 30% (Ahmed et al., 2022). Livestock serve as amplifying hosts but are typically asymptomatic. High-risk groups include abattoir workers, herders, and healthcare providers. In addition, frequent transboundary movement of livestock increases the risk of regional spread. Despite recurrent outbreaks, diagnostic and surveillance capacity in Sudan remains limited, and underreporting is likely (Ahmed et al., 2020b).

Serological and outbreak evidence of CCHFV has been reported in cattle, camels, and humans across multiple states, including East Darfur (Ibrahim et al., 2015; Bower et al., 2019), Kassala (Rahden et al., 2019), Khartoum (Suliman et al., 2017), North Kordofan (Adam et al., 2013), South Darfur (Ahmed et al., 2022), South Kordofan (Abdelhakam and Taha, 2014), Northern, and West Kordofan (Ahmed et al., 2020b) (Figure 6).

### 5.5 Q fever

Q fever is a zoonotic disease caused by the obligate intracellular bacterium *Coxiella burnetii*, which is highly resilient to environmental stress and capable of surviving in aerosols and dust for extended periods (España et al., 2020). The primary route of human infection is inhalation of contaminated aerosols or dust particles generated from birth products, placenta, urine, feces, or other excreta of infected animals (Kersh et al., 2013; Carugati et al., 2019). Although the role of ticks in the transmission of *C. burnetii* remains debated, there is evidence of bacterial detection in ticks, and experimental studies have demonstrated vector competence, including transstadial transmission across all *H. aegyptium* life stages (Široký et al., 2010; Körner et al., 2021).

In Sudan, serological evidence indicates circulation of *C. burnetii* in cattle, sheep, goats, and camels (Hussien et al., 2017). Human cases are rarely diagnosed, although undetected Q fever may contribute to febrile illnesses of unknown origin in pastoral communities (Vanderburg et al., 2014).

Clinically, Q fever in humans presents with fever, pneumonia, or hepatitis and may progress to chronic infection, including endocarditis. In livestock, infections are often subclinical but are associated with reproductive disorders such as abortion and infertility (Vanderburg et al., 2014).

Q fever has been reported across multiple regions in Sudan in cattle, camels, and goats, although most evidence is based on serological surveys. Documented findings span Al Gezira, Blue Nile, Kassala, Khartoum, North Darfur, North Kordofan, Northern State, River Nile, Sennar, South Darfur, South Kordofan, and White Nile states (Kirk, 1940; Hussien et al., 2012, 2017) (Figure 6). This wide geographical distribution suggests that *C. burnetii* is among the most widespread zoonotic pathogens associated with livestock and tick environments in Sudan. Given the country's reliance on small ruminants and camels, Q fever may have substantial but underrecognized socioeconomic impacts.

### 5.6 Ehrlichiosis

Ehrlichiosis is caused by obligate intracellular bacteria of the genus *Ehrlichia*. In Sudan, *E. ruminantium*, the causative agent of heartwater disease, is a major concern for livestock and is primarily transmitted by *Amblyomma* ticks. Heartwater is highly fatal in cattle, sheep, and goats, particularly in imported breeds (Muramatsu et al., 2005).

Clinical signs include high fever, neurological manifestations, hydropericardium, and sudden death. The disease is widely distributed across Sudan's savannah zones, where *A. variegatum* is prevalent. In humans, evidence of exposure to *E. chaffeensis* and related species has been reported; however, clinical disease remains poorly characterized (Muramatsu et al., 2005). The persistence of heartwater disease significantly hampers livestock development initiatives in Sudan by limiting the introduction and productivity of improved breeds (Latif, 1987).

*Ehrlichia* pathogens have been reported in sheep, goats, camels, cattle, and dogs across multiple regions in Sudan, including Al Gedarif (Karrar, 1960; Muramatsu et al., 2005), Al Gezira (Karrar, 1960; Latif, 1987; Suliman, 2011; Ibrahim et al., 2013), Blue Nile (Lee et al., 2018), East Darfur (Mossaad et al., 2021), Kassala (Karrar, 1960; Inokuma et al., 2006), Khartoum (Latif, 1987; Mossaad et al., 2021; Satti et al., 2021), North Darfur (Karrar, 1960), North Kordofan (Suliman, 2011), River Nile (Karrar, 1960), Sennar (Karrar, 1960; Jongejan et al., 1984; Latif, 1987; Saad, 2004; Muramatsu et al., 2005; Suliman, 2011; Mohammed et al., 2015), South Darfur (Abdalla, 2007; Suliman, 2011), South Kordofan (Sowar, 2002), West Kordofan (Suliman, 2011; Lee et al., 2018), and White Nile states (Karrar, 1960) (Figure 6).

### 5.7 Lumpy skin disease

Lumpy skin disease (LSD), caused by lumpy skin disease virus (a *Capripoxvirus*), is an economically devastating viral disease of cattle (Hussien et al., 2022). Although primarily transmitted mechanically by biting arthropods, ticks such as *Rhipicephalus* and *Amblyomma* species have also been implicated in transmission (Sprygin et al., 2019).

The disease is characterized by nodular skin lesions, fever, lymphadenitis, and secondary bacterial infections. Although mortality is generally low, morbidity is high, resulting in reduced milk production, infertility, and significant trade restrictions (Ali and Obeid, 1977). Sudan has experienced multiple LSD outbreaks in recent decades, affecting both local and crossbred cattle populations. The potential involvement of ticks as mechanical vectors highlights the complex transmission ecology of the disease in the country (Ali and Obeid, 1977).

LSD has been reported across several cattle-producing states in Sudan, including Al Gedarif, Al Gezira, Blue Nile, Kassala, Khartoum, Sennar, West Kordofan, and White Nile (Ali and Obeid, 1977; Saad, 2004; Hussien et al., 2022) (Figure 6). The widespread distribution of LSD across major livestock-producing regions underscores its importance as an endemic cattle disease, with ticks potentially contributing to transmission dynamics.

### 5.8 Rickettsioses

Ticks in Sudan harbor multiple spotted fever group *Rickettsia* species, including *R. africae*, the causative agent of African tick-bite fever (Nakao et al., 2015). Evidence of *Rickettsia* exposure has been reported in both humans and livestock, although confirmed clinical cases remain limited (Silva-Ramos and Faccini-Martínez, 2021). In humans, infection typically presents with fever, rash, and an eschar at the bite site. However, due to clinical overlap with other febrile illnesses such as malaria, rickettsioses are frequently misdiagnosed (Silva-Ramos and Faccini-Martínez, 2021). In livestock, infections remain poorly characterized, although ticks are recognized as both reservoirs and vectors of *Rickettsia* species (Nakao et al., 2015). The extensive tick diversity in Sudan suggests that rickettsioses may be underrecognized contributors to febrile illness in the country (Springer et al., 2020).

Rickettsial infections have been detected in ticks collected from cattle, sheep, and goats, as well as in tick species including *H. dromedarii, H. anatolicum*, and *H. rufipes* in Khartoum State (Morita et al., 2004; Eisawi et al., 2017, 2024). Evidence of tick-associated *Rickettsia* species, including *R. aeschlimannii, R. africae, R. raoultii, R. massiliae*, and *R. rhipicephali*, has been reported from West Darfur (Shuaib et al., 2020a), Al Gedarif (Morita et al., 2004; Nakao et al., 2015), Al Gezira (Shuaib et al., 2020a), Kassala (Morita et al., 2004; Springer et al., 2020), North Kordofan (Nakao et al., 2015; Springer et al., 2020), River Nile (Shuaib et al., 2020a), Sennar, South Kordofan, and White Nile state (Nakao et al., 2015) (Figure 6).

### 5.9 Theileriosis

Theileriosis is one of the most important livestock diseases in Sudan, caused by protozoa of the genus *Theileria* (Ali et al., 2017). The most pathogenic species is *T. annulata*, responsible for tropical theileriosis in cattle and transmitted by *Hyalomma* ticks. Other species, including *T. parva, T. mutans*, and *T. ovis*, have also been reported in the region (Gharbi et al., 2020).

Tropical theileriosis is characterized by fever, lymphadenopathy, anemia, and high mortality in susceptible cattle. It represents a major constraint to dairy and beef production, particularly in central and northern Sudan. Theileriosis remains a key priority in veterinary health policy due to its substantial economic burden (Mukhebi et al., 1992). Control strategies rely mainly on acaricide application and, in some areas, vaccination using attenuated schizont vaccines (Sharieff et al., 2017).

Reports of theileriosis in sheep, cattle, horses, and goats across Sudan indicate a wide geographical distribution, including Al Gedarif (Salih et al., 2009), Al Gezira (Latif, 1987; Saad, 2004; Salih et al., 2005, 2009; El Imam et al., 2016; Sharieff et al., 2017; Abakar et al., 2018; Hayati et al., 2020; Ibrahim et al., 2020; Magzoub et al., 2021; Abdelgalil et al., 2023), Blue Nile (Al Faki, 2004; El Hussein et al., 2004; Salih et al., 2009; El Imam et al., 2016; Lee et al., 2018), East Darfur (Mossaad et al., 2021), Kassala (Abdoon and Hussein, 1992; Imam, 1995; Salih et al., 2009; Ali et al., 2017; Hassan et al., 2019; Springer et al., 2020), Khartoum (Ibrahim et al., 2010; El Imam et al., 2016; Ali et al., 2017; Hassan et al., 2019; Mossaad et al., 2021), North Darfur (Salih et al., 2009), North Kordofan (Al Faki, 2004; Salih et al., 2009; Ali et al., 2017; Mohammed-Ahmed et al., 2018; Hassan et al., 2019; Springer et al., 2020; El Ayis, 2022), Northern (Ali et al., 2017; Hassan et al., 2019), Red Sea (Salih et al., 2009), River Nile (Salih et al., 2005, 2009; Taha et al., 2011, 2013; El Imam et al., 2016; Ali et al., 2017; Sharieff et al., 2017; Awad et al., 2018; Hassan et al., 2019), Sennar (Al Faki, 2004; Salih et al., 2009; Mohamed et al., 2018; Mohammed et al., 2018a), South Darfur (Adam, 2005; Abdalla, 2007; Salih et al., 2009; Gaafar et al., 2015; El Imam et al., 2016; Ali et al., 2017), South Kordofan (Sowar, 2002; Basheir et al., 2012), West Kordofan (Alkareem et al., 2012; Lee et al., 2018; Magzoub et al., 2021; El Ayis, 2022), and White Nile states (Al Faki, 2004; Saad, 2004; Salih et al., 2009; Guma et al., 2015; El Imam et al., 2016) (Figure 6). Its presence across nearly all livestock-intensive regions highlights its major role in cattle morbidity and mortality in Sudan.

### 5.10 Tick paralysis

Tick paralysis is a non-infectious, toxin-mediated clinical condition caused by neurotoxins secreted in the saliva of feeding *Hyalomma* or *Rhipicephalus* tick species (Mans et al., 2004). Although relatively rare, an outbreak has been reported in one-humped camels in South Darfur (Musa and Osman, 1990) (Figure 6).

Clinical signs include progressive ascending paralysis, typically beginning in the hind limbs and potentially progressing to respiratory failure (Grattan-Smith et al., 1997). Removal of the attached tick usually leads to rapid clinical recovery. Although its direct burden is lower than that of infectious TBDs, tick paralysis highlights the broader health risks associated with heavy tick infestations in Sudan (Musa and Osman, 1990). Tick toxicosis, therefore, remains an important but often underemphasized veterinary condition in the country.

### 5.11 Animal trypanosomiasis

Although animal trypanosomes are primarily transmitted by tsetse flies, some species, including *Trypanosoma theileri, T. vivax*, and *T. evansi*, can also be transmitted mechanically by ticks such as *H. anatolicum* and *R. pulchellus* (Burgdorfer et al., 1973; Shastri and Deshpande, 1981; Kernif et al., 2024). In Sudan, animal trypanosomiasis (surra, caused by *T. evansi*) is a major constraint on camel production, while *T. vivax* and *T. congolense* affect cattle and small ruminants (Mossaad et al., 2020).

Infected animals typically exhibit fever, anemia, emaciation, and reduced productivity. The disease is particularly prevalent in Blue Nile, Upper Nile, and southern regions of Sudan (Mossaad et al., 2020). Mechanical transmission by ticks and biting flies complicates control efforts outside the tsetse belt, and trypanosomiasis contributes substantially to economic losses, particularly in pastoral communities (Ahmed et al., 2016).

Evidence of trypanosomes associated with ticks, particularly *H. anatolicum*, has been reported in Sudan, including *T. theileri, T. vivax*, and *T. evansi* detected in camels, cattle, and horses from Al Gedarif (Bala et al., 2018c), Al Gezira (Morzaria et al., 1986; Latif, 1987), Blue Nile (Rahman, 2005), Kassala (Rahman, 2005), Khartoum (Morzaria et al., 1986), Sennar (Latif, 1987), South Darfur (Adam, 2005), and White Nile states (Saad, 2004) (Figure 6). While transmission is classically associated with tsetse flies, evidence suggests that ticks may play a role as mechanical vectors or alternative transmission facilitators in tsetse-free areas. This is supported by observations from regions such as Turkey and several European countries, where tsetse flies are absent but animal trypanosomiasis—particularly *T. theileri* infections—persists (Magri et al., 2021; Sahin et al., 2025).

### 5.12 Tularemia

Tularemia, caused by the bacterium *Francisella tularensis*, has been reported in parts of North Africa and the Middle East (Sholeh et al., 2024). Ticks, particularly *D. variabilis, D. andersoni*, and *A. americanum*, are important vectors, although transmission may also occur through direct contact with infected wildlife or contaminated water (Genchi et al., 2015; Maurin and Gyuranecz, 2016).

In humans, tularemia typically presents with fever, ulceroglandular lesions, and lymphadenitis (Sholeh et al., 2024). In Sudan, it has been reported only once in Khartoum, and confirmed outbreaks remain scarce (Mohamed et al., 2012) (Figure 6). However, the presence of suitable ecological conditions and competent tick vectors suggests that cases may be underdiagnosed. Limited surveillance and diagnostic capacity further obscure the true burden of disease. This restricted documentation likely reflects under-recognition rather than absence, underscoring the need for increased vigilance for this zoonotic pathogen in Sudan.

### 5.13 Dermatophilosis

Dermatophilosis is a bacterial skin disease of cattle, sheep, and goats caused by *Dermatophilus congolensis* (Gitao, 1992). Although it is not strictly a tick-borne infection, bites from *A. variegatum* can facilitate disease development by causing skin trauma and introducing immunomodulatory substances in tick saliva, which reduce host immune responses and promote bacterial invasion and establishment (Rodrigues et al., 2018). In Sudan, dermatophilosis is particularly common during the wet season and in regions with high tick burdens (Bakhiet et al., 2013).

Affected animals develop exudative dermatitis characterized by scab formation, alopecia, and reduced skin quality. Severe cases may progress to emaciation and secondary infections (Bakhiet et al., 2013). Economic impacts arise from hide damage, reduced market value, and decreased productivity. The disease also has zoonotic potential, occasionally affecting humans in close contact with infected animals (Zaria, 1993; Hamid and Musa, 2009).

Cases of dermatophilosis have been documented in camels and cattle in Al Gedarif, Al Gezira (Abakar, 2017), Khartoum (Bakhiet et al., 2013), and South Darfur states (Hamid and Musa, 2009) (Figure 6). Although not as geographically widespread as some protozoan infections, the disease remains important in areas where humid conditions and tick infestations predispose livestock to skin trauma and secondary bacterial invasion.

## 6 Driving factors for the expansion in the geographical distribution of ticks and TBDs

Changes in land use and land cover, including deforestation and unplanned urbanization, alongside the dynamics of large human and animal populations—including migratory birds—increased global trade and travel, and climate change are major drivers of shifts in the local composition of the tick populations and the pathogens they carry (Medlock et al., 2013; Berthová et al., 2016; Gilbert, 2021).

Collectively, these factors increase contact between humans, animals, and ticks, creating conditions that favor tick survival, long-distance passive dispersal, and the establishment of tick populations in new environments. They also enhance environmental suitability for local tick establishment and expansion (Estrada-Peña, 2001; Dantas-Torres, 2015; Selmi et al., 2018; Gilbert, 2021; Ogden et al., 2021; Lee and Chung, 2023; Cao et al., 2025).

## 7 Multisectoral One Health strategy for preparedness, prevention, and control of ticks and TBDs

Considering that ticks are widely distributed across diverse environments and ecological niches, and readily feeding on humans, domestic animals, and wildlife, as well as using animal movement and migratory birds for passive dispersal, a cost-effective, community-led, multisectoral One Health strategy is needed for the prevention and control of ticks and TBDs (Dantas-Torres et al., 2012; Inci et al., 2016; Stafford et al., 2017; Charles et al., 2021). In Sudan, elements of a One Health approach are present but remain largely fragmented, with activities often implemented independently across the human health, veterinary, and environmental sectors. Existing efforts are primarily embedded within livestock health programs, outbreak response initiatives, and limited vector surveillance activities, with minimal formal integration into a unified national One Health framework (Ahmed et al., 2024; Ali et al., 2024; Mohamed et al., 2025).

Key challenges to effective implementation include weak cross-sectoral coordination, limited institutionalization of One Health governance structures, and insufficient integration of surveillance systems across human and animal health sectors. In addition, diagnostic capacity for TBDs remains limited, particularly in peripheral and rural settings, constraining timely detection and response. Surveillance efforts are further hindered by inconsistent funding, reliance on short-term research projects, and lack of standardized methodologies for tick collection and pathogen detection. Other barriers include inadequate workforce capacity in medical and veterinary entomology, low community awareness of TBD risks, and limited incorporation of environmental and ecological data into public health decision-making. Political instability, competing public health priorities, and constrained resources further complicate sustained implementation of coordinated One Health strategies (Ahmed et al., 2025a,b,c).

Despite these challenges, several strengths provide a strong foundation for advancing One Health implementation in Sudan. These include an established network of veterinary services and livestock health programs, existing academic and research institutions with expertise in parasitology and vector-borne diseases, and prior experience with integrated approaches for zoonotic and epidemic-prone diseases. Community-based health systems and increasing regional and international collaborations also offer opportunities to support integrated surveillance and intervention strategies. These existing capacities can be leveraged to build scalable, coordinated, and cost-effective One Health programs (Cudjoe and Amin, 2024).

Strategic One Health interventions should therefore focus on strengthening integrated surveillance systems, expanding diagnostic capacity in remote settings, and improving coordination across sectors (Ahmed et al., 2021; Mohamed et al., 2024a,b). This includes integrating molecular diagnostics into regional laboratories, training frontline health workers to recognize and promptly report TBDs, and establishing coordinated surveillance frameworks linking human, animal, and environmental data streams, consistent with guidance from organizations such as the WHO and WOAH can reduce duplication of effort while improving early detection and response. Leveraging cross-sectoral activities, such as training entomologists and veterinary personnel to collect tick samples during routine field activities can substantially improve surveillance coverage while minimizing costs (Banović et al., 2021; Johnson et al., 2022).

Additionally, a central pillar of a fully functional One Health approach is strong community engagement. Effective health education and behavior-change strategies empower communities to take ownership of their health and implement sustainable, community-led interventions (Ahmed et al., 2020c; Ali et al., 2022; Mohamed et al., 2025; Ngabonziza et al., 2026). Strengthening health education and behavior-change strategies can empower communities to adopt preventive measures, including personal protection during peak tick seasons, improved environmental hygiene, and better management of animal shelters. Additional interventions include landscape management; reducing contact between domestic animals and wildlife, and the appropriate use of repellents and acaricides (Dantas-Torres et al., 2012; Ajith Kumar et al., 2016; Laing et al., 2018). In parallel, emerging tick-control strategies offer promising additions to integrated control frameworks. These include anti-tick vaccines, ivermectin-based systemic control approaches, and advancing *in vitro* and *in silico* research targeting both vector and pathogen components. Notably, progress in human vaccines development, particularly next-generation Lyme disease candidates, alongside experimental anti-tick antigen platforms, highlights the potential for innovative, long-term solutions (Park et al., 2019; Marconi et al., 2020; Muhanguzi et al., 2022; Sarli et al., 2022; Abbas et al., 2023; Mohamed et al., 2026). Collectively, these coordinated public health and One Health actions represent essential measures for strengthening sustainable prevention, surveillance, and control of ticks and TBDs.

## 8 Conclusions

TBDs in Sudan represent a major challenge at the interface of human, animal, and environmental health. Livestock diseases such as theileriosis, babesiosis, anaplasmosis, and lumpy skin disease cause substantial economic losses, undermining food security and constraining economic development. Zoonotic infections—including CCHF, borreliosis, Q fever, ehrlichiosis, rickettsioses, and possibly tularemia—pose additional threats to public health, and are often underrecognized due to limited laboratory diagnostic capacity and the predominance of malaria in the differential diagnosis of febrile illness. The persistence of these diseases in Sudan reflects the abundance of competent tick vectors, high livestock mobility, weak veterinary infrastructure, and limited public health surveillance. Addressing this burden requires integrated, cost-effective, multisectoral One Health approaches, including strengthened vector surveillance and control, improved diagnostic and surveillance systems, vaccination where available, improved hygiene and landscape management, and enhanced cross-border collaboration. Implementing One Health approaches is essential to mitigate the combined human and animal health impacts of TBDs in Sudan.
